# Safety of Alemtuzumab and Autologous Hematopoietic Stem Cell Transplantation Compared to Noninduction Therapies for Multiple Sclerosis

**DOI:** 10.1212/WNL.0000000000011545

**Published:** 2021-03-16

**Authors:** Peter Alping, Joachim Burman, Jan Lycke, Thomas Frisell, Fredrik Piehl

**Affiliations:** From the Department of Clinical Neuroscience (P.A., F.P.) and Clinical Epidemiology Division (P.A., T.F.), Department of Medicine Solna, Karolinska Institutet, Stockholm; Department of Neuroscience (J.B.), Uppsala University; Department of Clinical Neuroscience (J.L.), Institute of Neuroscience and Physiology, University of Gothenburg; and Academic Specialist Centre (F.P.), Stockholm Health Services, Sweden.

## Abstract

**Objective:**

To assess safety outcomes for the induction therapies alemtuzumab and autologous hematopoietic stem cell transplantation (AHSCT) compared to noninduction disease-modifying therapies.

**Methods:**

We performed a population-based cohort study linking the Swedish Multiple Sclerosis Register to national health care registers. Alemtuzumab, AHSCT, and a matched reference group of noninduction therapies (natalizumab, dimethyl fumarate, rituximab, fingolimod) were included if started between 2008 and 2017. Main outcomes were death, thyroid disease, nonthyroid autoimmune disease, and infection.

**Results:**

We identified 132 alemtuzumab-treated and 139 AHSCT-treated (68% high-dose cyclophosphamide and anti-thymocyte globulin [ATG], 32% BCNU, etoposide, cytosine-arabinoside, and melphalan/ATG) patients, together with 2,486 matched patients treated with noninduction therapies. Four patients in the alemtuzumab group died (incidence rate [IR] per 1,000 person-years 8.6, 95% confidence interval [CI] 2.3–22.0) compared to 1 patient in the AHSCT group (IR 1.7, 95% CI 0.0–9.6), and the mortality rate in the reference group was 0.7 (95% CI 0.3–1.3). Thyroid disease was most frequent in the alemtuzumab group (IR 109, 95% CI 75–154) but also occurred more often for AHSCT (IR 34, 95% CI 18–56) compared to the reference (IR 5.3 95% CI 3.9–7.1). The incidence of nonthyroid autoimmune disease was similar in all groups. IR for infection diagnosed ≥6 months from therapy initiation was 53 (95% CI 30–87) for alemtuzumab, 108 (95% CI 75–150) for AHSCT, and 51 (95% CI 46–57) for the reference.

**Conclusion:**

We confirmed a high incidence of thyroid disease in alemtuzumab- and, to a smaller extent, AHSCT-treated patients and found a higher incidence of infection for AHSCT compared to both alemtuzumab and noninduction therapies. The incidence of nonthyroid autoimmune disease was low for both therapies.

**Classification of Evidence:**

This study provides Class III evidence of an increased risk of thyroid disease with alemtuzumab and an increased risk of infection with AHSCT treatment.

Disease-modifying therapies (DMTs) for multiple sclerosis (MS) can be classified by type of administration: repeated administration vs induction-type therapies that cause long-term cessation of MS-associated inflammation. Alemtuzumab was the first induction-type therapy to be approved for relapsing-remitting MS (RRMS); it was approved in 2013 by the European Medicines Agency and 2014 by the US Food and Drug Administration. Autologous hematopoietic stem cell transplantation (AHSCT) is another induction-type medical procedure that has been used to treat MS since 1995^1^ and was approved for use in active RRMS in Sweden in 2016.

Alemtuzumab is a humanized monoclonal antibody that induces depletion and subsequent repopulation of CD52+ immune cell populations, leading to long-lasting changes to adaptive immunity.^2^ It is administered as daily 12-mg IV infusions over 5 days, with 3 additional infusions after 1 year and further cycles as needed.

The AHSCT procedure consists of hematopoietic stem cell (HSC) mobilization, HSC harvesting, conditioning, and HSC transplantation.^3^ The 2 main conditioning regimens are high-dose cyclophosphamide (Cy) and anti-thymocyte globulin (Cy/ATG) or a combination of 4 cytostatic agents (BCNU, etoposide, cytosine-arabinoside, and melphalan; BEAM) and ATG.

In Sweden, alemtuzumab and AHSCT have been used primarily for aggressive RRMS or breakthrough inflammation on conventional therapies. Preference for either therapy has been driven mainly by local experience and availability. Widespread use of these therapies has been limited by concerns and uncertainty concerning their respective safety profiles in relation to other MS therapies.

The aim of this study was to describe safety outcomes after treatment with alemtuzumab and AHSCT compared to noninduction therapies.

## Methods

We performed a nationwide register-based cohort study of alemtuzumab and AHSCT compared to matched noninduction therapies, linking data from the Swedish MS register to national health care and demographic registers.

### Data Sources

The nationwide Swedish MS register captures data on several aspects of MS care^4^ and has especially high validity for therapy data.^5^ Participation is voluntary, but coverage has been estimated to be >80%.^6^ The MS register was linked to several national registers with compulsory participation: the cause of death register, the patient register (all visits to inpatient and specialized outpatient care and their associated diagnosis codes, with high validity),^7^ the prescribed drug register (complete data on all prescription drugs collected at pharmacies),^8^ the cancer register,^9^ demographic registers, and registers with data on sick leave and disability pension. The end of data collection differed between the registers; the MS, cause of death, and prescribed drug registers had data until September 30, 2018, and the patient and the cancer registers had data until December 31, 2017.

### Exposure Definition

We included all first-ever treatments with alemtuzumab and AHSCT, as well as a reference group of 4 noninduction therapies, registered in the MS register and started between January 1, 2008, and December 31, 2017. The noninduction therapies comprised rituximab, fingolimod, natalizumab, and dimethyl fumarate, the 4 most frequently used MS DMTs in Sweden. AHSCT treatments were grouped according to the conditioning regimen used, Cy/ATG or BEAM/ATG. Previous treatment with alemtuzumab or AHSCT was not allowed, and patients were censored on death, emigration, or start of a subsequent treatment with alemtuzumab or AHSCT or at end of data collection, whichever happened first. Patients were excluded if they were <15 years of age at therapy start.

### Outcome Definition and Baseline Variables

Primary outcomes were death, thyroid disease, nonthyroid autoimmune disease, and infection. Deaths were gathered primarily from the cause of death register, but the cause was not available for all observations (due to registry-entry delay), and supplementary information reported directly to the Swedish Medical Products Agency was used in these cases. Thyroid disease, nonthyroid autoimmune disease, and infections were identified by ICD codes (table 1) from the primary and secondary diagnoses of the inpatient and specialized outpatient visits in the patient register. For thyroid disease and nonthyroid autoimmune disease, only the first-ever event was included, and if this event happened before therapy start, the patient was censored. To also catch thyroid disease treated in primary care, thyroid hormone prescriptions were identified by Anatomical Therapeutic Chemical Classification System (ATC) codes from the prescribed drug register (table 1). Several other outcomes were also investigated according to what had been previously reported: cancer, blood cell disorders (aplastic anemia, thrombocytopenia, agranulocytosis), disorders of the reproductive organs and fertility, and starting a new MS DMT. The outcomes were identified through their respective registers (ICD and ATC-based definitions in table 1). For ATC-based outcomes, a drug was considered a new treatment and included only if there had been no registration of that particular drug in the previous 180 days. The baseline variables age, sex, birthplace, year, region, and MS type/onset/duration were assessed at therapy start. Education level, therapy count, thyroid disease, and nonthyroid autoimmune disease were measured from start of follow-up until start of therapy. Hospital days were counted from 5 years before until 1 month before therapy start. Disability pension and sick leave were counted in the year before therapy start. Expanded Disability Status Scale (EDSS) score was assessed 6 months before until 1 month after therapy start, and Symbol Digit Modalities Test (SDMT) and Multiple Sclerosis Impact Scale (MSIS-29) scores were obtained 3 months before until 1 month after therapy start.

**Table 1:**
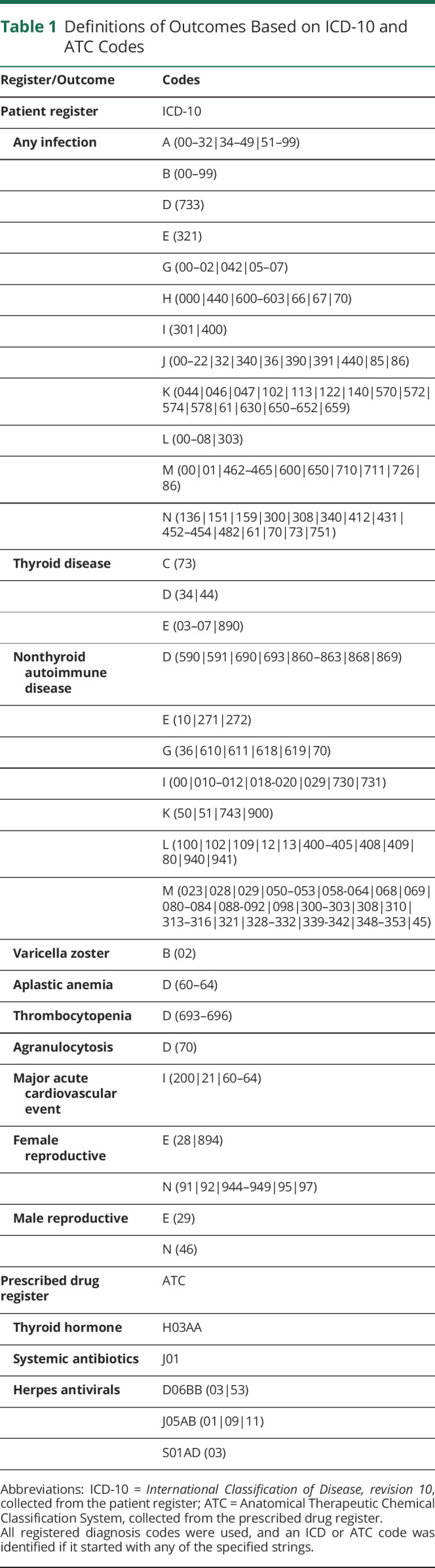
Definitions of Outcomes Based on ICD-10 and ATC Codes

### Analysis

The main focus of this study was to provide descriptive statistics for each cohort, rather than adjusted tests of group differences, because of the low numbers of treated patients. Incidence rates (IRs) per 1,000 person-years with 95% confidence intervals (CIs) were calculated for all outcomes. Kaplan-Meier plots were produced for the outcomes first-ever thyroid disease and starting a new therapy. The monthly IRs 3 years before and after therapy start were plotted in 3-month intervals for infection and collection of prescription drugs. Similarly, the proportion of time spent in inpatient care was plotted for the same intervals. The noninduction reference group was matched 10:1 to the main exposure groups by age, sex, and region using incidence-density sampling, and individuals were eligible to be selected as referents if at the start of noninduction therapy they had not previously been treated with alemtuzumab or AHSCT. To investigate nonimmediate effects of the therapies, a lag time of 6 months from therapy initiation to when follow-up time started was used for the outcomes: infection, aplastic anemia, thrombocytopenia, and agranulocytosis.

All statistical analyses were done with Python 3.7.4 (Python^10^ Software Foundation, Wilmington, DE).

### Standard Protocol Approvals, Registrations, and Patient Consents

This study was approved by the Regional Ethical Board of Stockholm (reference 2017/700–31/4).

### Data Availability

Data are available on approval from the respective register holders.

## Results

We identified 132 alemtuzumab-treated and 139 AHSCT-treated (68% Cy/ATG, 32% BEAM/ATG) patients with mean follow-up of 3.5 and 4.2 years, respectively, in analyses of death, new therapy, and prescription drug–based outcomes and 2.8 and 3.4 years, respectively, for the diagnosis-based outcomes and cancer. The 2,486 noninduction therapies (36% natalizumab, 29% dimethyl fumarate, 22% rituximab, 13% fingolimod) were matched to the alemtuzumab and AHSCT therapies in a reference group with a mean follow-up of 4.3 and 3.6 years, depending on the outcome.

### Patient Characteristics

Patients in the alemtuzumab group were on average 2 years older with a lower proportion of women and a lower level of education compared to the patients in the AHSCT group (table 2). Patients receiving alemtuzumab also had less hospital-admission time, disability pension, sick leave, and autoimmune disease; later onset of MS; shorter disease duration; fewer previous therapies; lower (i.e., better) EDSS and MSIS-29 scores; and higher (i.e., better) SDMT scores. Within the AHSCT group, patients receiving Cy/ATG were slightly younger; were more often female and with the RRMS type; had lower education, less hospital admission time, less sick leave, and less nonthyroid autoimmune disease, and lower age at MS onset; more often had a single previous therapy; and had lower EDSS and MSIS-29 scores compared to the patients receiving BEAM/ATG. There were major regional differences in therapy choice, with most regions showing a preference for either alemtuzumab or AHSCT and for 1 of the 2 conditioning regimens used for AHSCT (table 2). Before matching, patients starting reference treatments were 5 to 7 years older than the patients in the alemtuzumab and AHSCT groups, had less hospital days and sick leave, and had tried fewer previous therapies. These differences were less obvious in the age-, sex-, and region-matched reference group. Data were missing for some of the baseline variables, and the missingness varied between the treatment groups, but there were no missing observations for any of the outcome variables. Most missing data involved baseline EDSS, SDMT, and MSIS-29 scores, especially for AHSCT-treated patients, among whom 22% to 81% of patients had missing data (table 3).

**Table 2:**
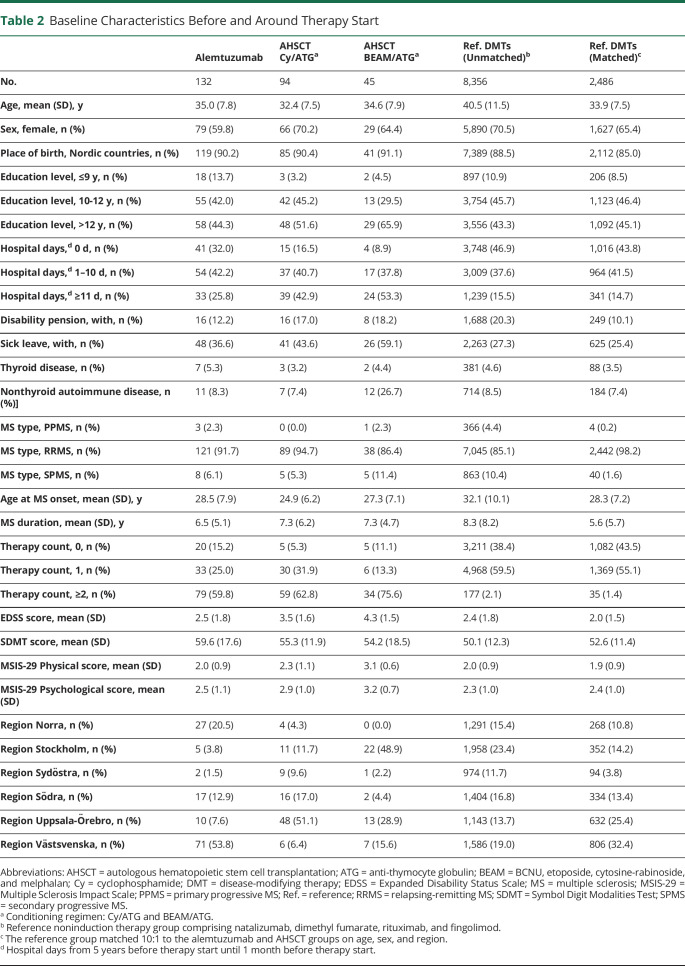
Baseline Characteristics Before and Around Therapy Start

**Table 3:**
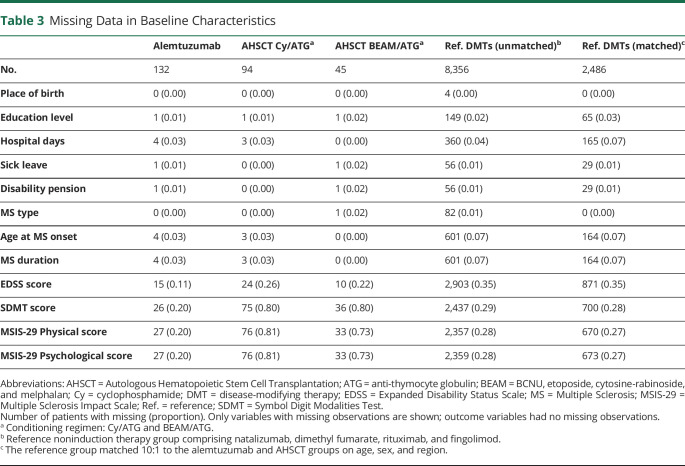
Missing Data in Baseline Characteristics

### Mortality

There were 4 deaths recorded in the alemtuzumab group (IR per 1,000 person-years 8.6, 95% CI 2.3–22.0, table 4): 2 suicides, 1 heart attack (1.4 years after therapy start), and 1 cytomegalovirus reactivation with multiorgan failure within 1 month of therapy start. There was 1 death, a suicide, reported in the AHSCT group (BEAM/ATG, >6 years after therapy start), corresponding to an IR of 1.7 (95% CI 0.0–9.6). The mortality rate in the matched reference group was 0.7 (95% CI 0.3–1.3).

**Table 4:**
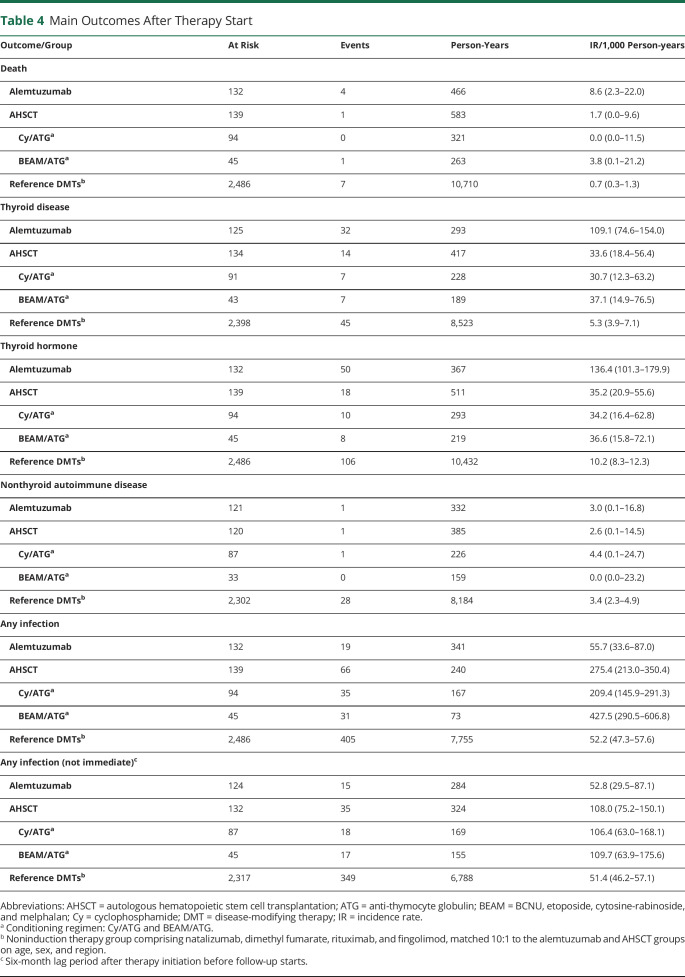
Main Outcomes After Therapy Start

### Thyroid Disease and Nonthyroid Autoimmune Disease

**Figure 1:**
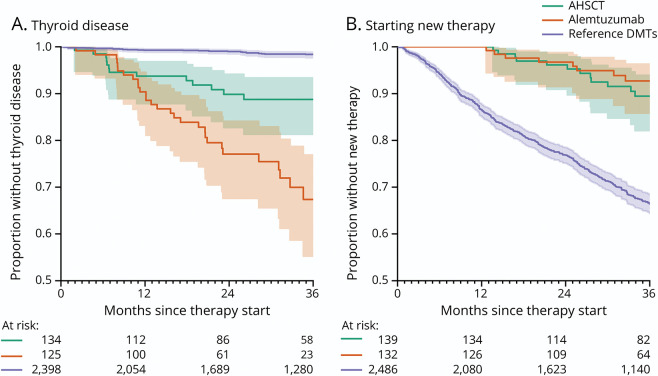
Thyroid Disease After Therapy Start and Proportion of Patients Not Starting a New Therapy Kaplan-Meier plots of (A) proportion of patients without thyroid disease after therapy start and (B) proportion of patients not starting a new therapy. Patients with thyroid disease before therapy start were censored. Reference group comprised the noninduction therapies natalizumab, dimethyl fumarate, rituximab, and fingolimod, matched 10:1 to the alemtuzumab and autologous hematopoietic stem cell transplantation (AHSCT) groups on age, sex, and region. DMT = disease-modifying therapy.

A first-ever thyroid disease was diagnosed in inpatient or specialized outpatient care in 32 alemtuzumab- and 14 AHSCT-treated patients (IRs per 1,000 person-years 109 [95% CI 75–154] and 34 [95% CI 18–56], respectively, table 4). The corresponding IR for the matched reference group was 5.3 (95% CI 3.9–7.1). A similar pattern was evident for prescriptions of thyroid hormone, which in addition captures thyroid disease managed in primary care (table 4). Figure 1 (left panel) shows the proportion of patients without a diagnosis of thyroid disease over time from therapy start. First-ever nonthyroid autoimmune disease was rare, with an IR 3.0 (95% CI 0.1–16.8) for alemtuzumab, 2.6 (95% CI 0.1–14.5) for AHSCT, and 3.4 (95% CI 2.3–4.9) for the matched reference (table 4).

### Infection

**Figure 2:**
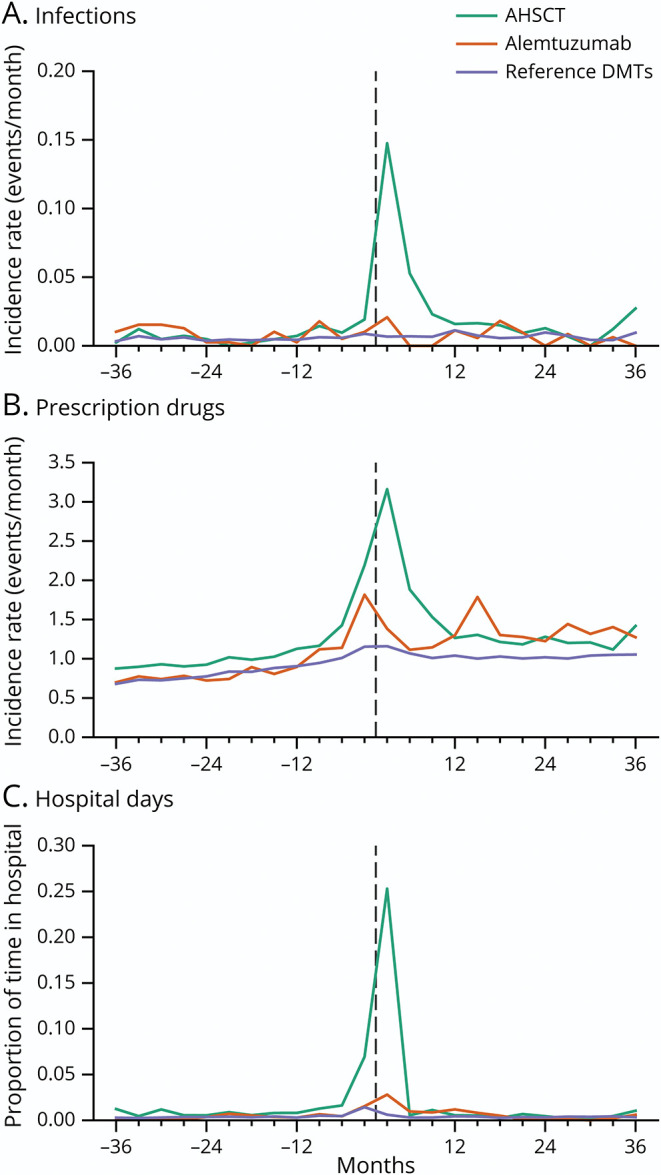
Infections, Drug Prescriptions, and Time Spent in Inpatient Care Before and After Therapy Start Incidence rate (events per month) of (A) diagnosed infection and (B) prescription of drugs and (C) the proportion of time spent in inpatient care in 3-month intervals 3 years before and after therapy start. Patients were not censored at an event and continued to contribute person-time. Reference group comprised the noninduction therapies natalizumab, dimethyl fumarate, rituximab, and fingolimod, matched 10:1 to the alemtuzumab and autologous hematopoietic stem cell transplantation (AHSCT) groups on age, sex, and region. DMT = disease-modifying therapy.

An infection diagnosed in inpatient or specialized outpatient care occurred in 19 alemtuzumab- and 66 AHSCT-treated patients (IR per 1,000 person-years 56 [95% CI 34–87] and 275 [95% CI 213–350], respectively, table 4). The corresponding IR for the matched reference group was 52 (95% CI 47–58). The incidence of infection was highest immediately after AHSCT but dropped to a level closer to the alemtuzumab and reference groups within the first year (figure 2). No clear temporal pattern could be discerned in the alemtuzumab and reference groups. When we assessed the nonimmediate effects of therapy by starting follow-up 6 months after therapy initiation, the IR for infection was instead 53 (95% CI 30–87) for alemtuzumab, 108 (95% CI 75–150) for AHSCT, and 51 (95% CI 46–57) for the matched reference (table 4). The rate of infection differed between the AHSCT conditioning regimens, with an initial higher rate for BEAM/ATG (IR 428, 95% CI 291–607) compared to Cy/ATG (IR 209, 95% CI 146–291). However, this difference disappeared after 6 months of follow-up. The most commonly diagnosed infections close to AHSCT treatment were herpesvirus infections (especially varicella zoster and in the BEAM/ATG group) and bacterial sepsis, followed by a wide variety of both viral and bacterial infections (data not shown). After 6 months, herpes infections were still the most commonly diagnosed infectious event, especially in the BEAM/ATG subgroup. For alemtuzumab, infections were more heterogeneous. Diagnosis of varicella zoster infection had an IR 8.3 for alemtuzumab, 20.4 for AHSCT (Cy/ATG 16.6, BEAM/ATG 24.9), and 1.8 for the matched reference (table 5). Systemic antibiotics and herpes antivirals were given to almost all patients after AHSCT and to a majority of patients after alemtuzumab (table 5). No cases of progressive multifocal leukoencephalopathy were found.

**Table 5:**
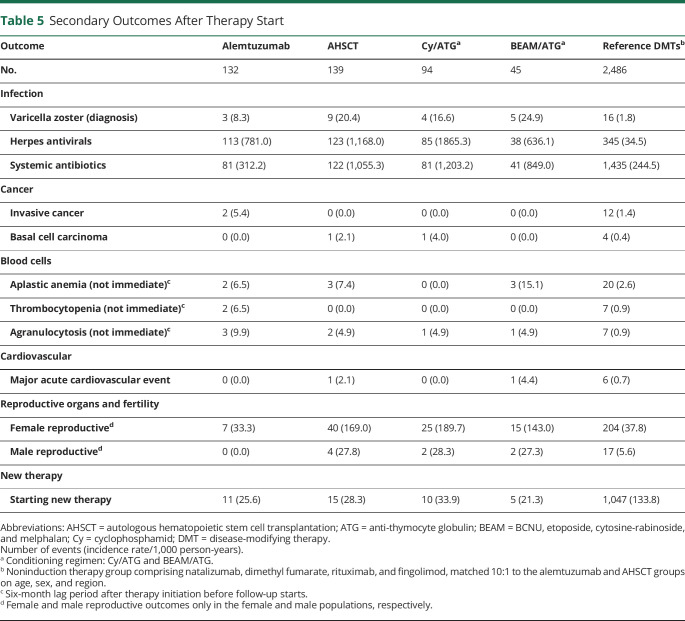
Secondary Outcomes After Therapy Start

### Other Safety Outcomes

Two cases of invasive cancer were recorded in the alemtuzumab group (IR per 1,000 person-years 5.4, breast and urinary bladder cancer, respectively) compared to no cancers in the AHSCT group and an IR of 1.4 in the matched reference (table 5). One case of basal cell carcinoma was reported in the AHSCT group (table 5). Blood cell disorders (aplastic anemia, thrombocytopenia, and agranulocytosis) were rare when follow-up was started after a lag time of 6 months from therapy initiation (table 5). There were no major acute cardiovascular events in the alemtuzumab group and 1 event in the AHSCT group (a cerebral infarction) (table 5). Diagnoses relating to the reproductive organs and fertility were common with AHSCT, especially in women (IR 169) and in the Cy/ATG subgroup (IR 190), compared to the other groups (IR 33–38) (table 5).

### Starting New Therapy

The IRs per 1,000 person-years for starting a new MS DMT were 26 (95% CI 13–46) for alemtuzumab and 28 (95% CI 16–47) for AHSCT, which were substantially lower than for the matched reference group (IR 134, 95% CI = 126–142) (table 5). Patients with AHSCT having received the Cy/ATG conditioning regimen had a higher rate of starting a new therapy compared to those receiving BEAM/ATG. Figure 1 (right panel) shows the proportion of patients without new therapy over time from therapy start.

### Prescription Drugs and Health Care

Drug prescriptions increased slightly over time in all groups, with peaks around therapy start (highest for AHSCT) and the second dosing of alemtuzumab after 1 year (figure 2). AHSCT was also associated with considerable in-hospital care, corresponding to 25% of all days in the first 3 months after therapy start (figure 2). This pattern was less evident for alemtuzumab and the noninduction therapies, and hospitalizations stabilized at <1% of the total time in all groups after the first 3 months of therapy.

## Discussion

In this observational study using high-quality data from clinical practice recorded in Sweden's nationwide health registers, we investigated safety outcomes for all patients with MS in Sweden treated with alemtuzumab and AHSCT compared to a matched reference group receiving noninduction MS DMTs. While the effectiveness of both alemtuzumab and AHSCT is considered high, safety concerns have limited their use, and studies comparing safety outcomes across therapeutic options have so far been rare. Our main findings were a higher incidence of thyroid disease with alemtuzumab and, to a lesser extent, with AHSCT, a higher incidence of infection with AHSCT, and a low incidence of nonthyroid autoimmune disease for both therapies.

A major concern with alemtuzumab treatment has been an increased risk of secondary autoimmune disease, in particular thyroid disease and immune-mediated thrombocytopenia.^2,11,12^ We report here a higher incidence of thyroid disease after treatment with alemtuzumab than previously seen in the clinical trials^2,11,12^ and in line with recent observational data.^13^ Notably, preexisting autoimmune conditions represented an exclusion criterion in the Comparison of Alemtuzumab and Rebif® Efficacy in Multiple Sclerosis (CARE-MS) trials.^2,11^ We further found a higher incidence of thrombocytopenia after alemtuzumab compared to the matched reference group, similar to what was found in the clinical trials.^2,11,12^ The incidence of first-ever nonthyroid autoimmunity with alemtuzumab was similar to that in the reference and AHSCT groups. Unfortunately, the groups were too small to analyze whether preexisting or posttreatment emerging thyroid autoimmunity was associated with a higher incidence of nonthyroid autoimmune disease after treatment. No cases of autoimmune hepatitis or hemophagocytic lymphohistiocytosis were recorded. The incidence of diagnosed infection in the alemtuzumab group was similar to that in the reference group, but the use of antibiotics and especially antivirals was higher after alemtuzumab, likely reflecting an actual or perceived increased infection risk that is managed prophylactically. One of the 4 deaths in the alemtuzumab group was directly related to the alemtuzumab treatment, a case of cytomegalovirus reactivation with multiple organ failure occurring within 1 month of the first treatment cycle. For the remaining 3 cases, the relation to alemtuzumab was uncertain.

A range of different treatment-related adverse events have been reported for AHSCT, of which treatment-related mortality is the most severe.^3^ In our study, only 1 death occurred after AHSCT during the observation period, a suicide 6 years after therapy start. The mortality rate for AHSCT was similar to that of the matched reference group and much lower than in most previous reports.^3^ This may be explained in part by the type of ablative conditioning used because less aggressive regimens have become more common. In this cohort, Cy/ATG had been used in 68% and BEAM/ATG in 32% of the patients undergoing AHSCT, and there were some differences in the baseline characteristics of these subgroups (table 2). A diagnosis of infection was the most common adverse event with AHSCT. The incidence was highest immediately after therapy start but remained higher than for both the alemtuzumab and the matched reference groups after 6 months from therapy start. The incidence of infection was higher close to BEAM/ATG conditioning compared to Cy/ATG but dropped to similar levels after 6 months. This difference between the conditioning regimens should be interpreted cautiously because routines for infection prophylaxis may have differed. According to previous studies, the infection risk in the reference group varies depending on type of DMT, with rituximab (22% of our reference group) having the highest risk of hospitalized infections.^14^ The incidence of thyroid disease in the AHSCT group was higher than in the matched reference group but lower than in the alemtuzumab group. Disorders relating to the reproductive organs and fertility were common in the AHSCT group, especially in women and in those having received Cy/ATG conditioning. The Cy/ATG subgroup was also more likely to start a new therapy compared to the BEAM/ATG subgroup.

The main strengths of this study are the use of national health registers with mandatory reporting and virtually complete coverage, together with the Swedish MS register with its high-quality data on treatment episodes and the availability of a large reference group of noninduction therapies. Although the data were of good overall quality, there was significant missingness in the rating scales of disease severity around treatment start, which was distributed unevenly across therapies. However, there were no missing observations for any of the outcome variables, and the missingness affected mainly the ability to perform more exact matching on disease characteristics. We also had no information on visits and diagnoses in general-practice outpatient care, and it is therefore likely that the incidences reported for the diagnosis-based outcomes are underestimated. This is a concern mainly for milder adverse events managed in primary care because more serious illness will be treated in inpatient or specialized outpatient care for which data were available. Because the follow-up ranged between 3 and 4 years, it was not possible to detect longer-term adverse events, which is especially important to consider when interpreting the incidence of cancer and serious late-onset autoimmune events. Additional limitations include potential channeling to therapy and surveillance bias, making direct comparisons between the groups more difficult. However, the regional preference and experience of treatment with either alemtuzumab or AHSCT seemed to be the decisive factor explaining the difference in treatment choice (table 2). The channeling to therapy effect would therefore be strongest in comparisons to the noninduction therapy reference group, for which matching was performed to mitigate this issue. It is likely that residual bias still exists in this comparison because the matching did not involve any MS-specific covariates and it is well accepted in the clinic that alemtuzumab and AHSCT are generally used to treat more aggressive disease, normally requiring breakthrough on previous therapies (as can be seen in the number of previous therapies in table 2). The difference in aggressiveness of MS would be more important if we were to investigate effectiveness outcomes, but the higher use of previous MS therapies could potentially also affect safety outcomes by a carryover effect, in which a previous therapy is actually causing the events that we find. However, because the majority of previous therapies are those in the reference group, any effect that could carry over to the alemtuzumab and AHSCT groups would also be seen in the reference group. A possible source of surveillance bias was that the patients undergoing AHSCT will generally be monitored more closely during and in the months after conditioning, often in the hospital, compared to those receiving alemtuzumab or the noninduction therapies. On the other hand, alemtuzumab-treated patients are followed up more carefully with blood and urine tests. These differences also complicate the severity assessment of infections, especially around therapy start for AHSCT because hospitalization occurs regardless. In addition, prophylactic antibiotics and antivirals are recommended for 6 to 12 months after AHSCT and antivirals for 1 month after alemtuzumab infusion, limiting the use of these as indicators of infectious events and potentially altering the comparison to the reference group for which there are no such recommendations. Finally, the preference of conditioning in the AHSCT group has changed over time, with a sharp increase of Cy/ATG in 2014 (data not shown). Because recommendations and guidelines for AHSCT treatment have likely also changed over the years, comparisons between the conditioning subgroups should be made with caution. Studies focused specifically on comparing these conditioning regimens are needed to explore any differences further.

Despite the descriptive nature of this study, the differences in incidence of thyroid disease and infection between the groups were great enough to allow a comparative interpretation even in the absence of confounder adjustment. There were differences in baseline characteristics between the groups, but they were mostly minor, and it is unlikely that confounding alone could cause the sharp increase in incidence that occurred directly after patients received treatment. The size of the differences and the clear temporal relationship with therapy start make a causal interpretation plausible.

A third induction-type therapy, cladribine, was approved for MS in 2017, but because it is so new, there were not enough treated patients to include it in our analyses. Future studies are needed to investigate how cladribine compares to alemtuzumab and AHSCT.

Collectively, in this study of 132 alemtuzumab- and 139 AHSCT-treated patients, we confirm the previously reported increased incidence of thyroid disease for alemtuzumab and, to a lesser extent, also for AHSCT and found a higher incidence of infection for AHSCT (highest for BEAM/ATG) compared to both the alemtuzumab group and the matched noninduction therapy reference group. The incidence of first-ever nonthyroid autoimmune events was similar between the groups. Disorders relating to reproductive organs and fertility were common in the AHSCT group, especially in women. Other adverse events were rare but still more common in the alemtuzumab and AHSCT groups than in the matched reference group. Overall mortality was slightly higher in the alemtuzumab group, but only 1 of the 4 deaths was clearly linked to the treatment.

## Glossary

AHSCT: autologous hematopoietic stem cell transplantation
ATC: Anatomical Therapeutic Chemical Classification System
ATG: anti-thymocyte globulin
BEAM: BCNU, etoposide, cytosine-arabinoside, and melphalan
CARE-MS: Comparison of Alemtuzumab and Rebif Efficacy in Multiple Sclerosis
CI: confidence interval
DMT: disease-modifying therapy
EDSS: Expanded Disability Status Scale
HSC: hematopoietic stem cell
ICD: *International Classification of Diseases*
IR: incidence rate
MS: multiple sclerosis
MSIS-29: Multiple Sclerosis Impact Scale
RRMS: relapsing-remitting MS
SDMT: Symbol Digit Modalities Test
